# Striking parallels between carotid body glomus cell and adrenal chromaffin cell development

**DOI:** 10.1016/j.ydbio.2018.05.016

**Published:** 2018-12-01

**Authors:** Dorit Hockman, Igor Adameyko, Marketa Kaucka, Perrine Barraud, Tomoki Otani, Adam Hunt, Anna C. Hartwig, Elisabeth Sock, Dominic Waithe, Marina C.M. Franck, Patrik Ernfors, Sean Ehinger, Marthe J. Howard, Naoko Brown, Jeffrey Reese, Clare V.H. Baker

**Affiliations:** a Department of Physiology, Development and Neuroscience, University of Cambridge, Anatomy Building, Downing Street, Cambridge CB2 3DY, United Kingdom; b Weatherall Institute of Molecular Medicine, John Radcliffe Hospital, Headley Way, Oxford OX3 9DS, United Kingdom; c Department of Molecular and Cell Biology, University of Cape Town, Cape Town, South Africa; d Department of Physiology and Pharmacology, Karolinska Institute, S-171 77 Stockholm, Sweden; e Center for Brain Research, Medical University Vienna, 1090 Vienna, Austria; f Institut für Biochemie, Emil-Fischer-Zentrum, Friedrich-Alexander-Universität Erlangen-Nürnberg, Fahrstrasse 17, 91054 Erlangen, Germany; g Wolfson Imaging Centre, Weatherall Institute of Molecular Medicine, John Radcliffe Hospital, Headley Way, Oxford OX3 9DS, United Kingdom; h Unit of Molecular Neurobiology, Department of Medical Biochemistry and Biophysics, Karolinska Institute, S-171 77 Stockholm, Sweden; i Department of Neurosciences and Program in Neurosciences and Neurodegenerative Diseases, University of Toledo Health Sciences Campus, Toledo, OH 43614, USA; j Depts. of Pediatrics, Cell and Developmental Biology, Vanderbilt University Medical Center, 2215 B Garland Avenue, Nashville, TN 37232, USA

**Keywords:** Carotid body glomus cells, Adrenal chromaffin cells, Neural crest, Schwann cell precursors, Nodose neurons

## Abstract

Carotid body glomus cells mediate essential reflex responses to arterial blood hypoxia. They are dopaminergic and secrete growth factors that support dopaminergic neurons, making the carotid body a potential source of patient-specific cells for Parkinson's disease therapy. Like adrenal chromaffin cells, which are also hypoxia-sensitive, glomus cells are neural crest-derived and require the transcription factors Ascl1 and Phox2b; otherwise, their development is little understood at the molecular level. Here, analysis in chicken and mouse reveals further striking molecular parallels, though also some differences, between glomus and adrenal chromaffin cell development. Moreover, histology has long suggested that glomus cell precursors are ‘émigrés’ from neighbouring ganglia/nerves, while multipotent nerve-associated glial cells are now known to make a significant contribution to the adrenal chromaffin cell population in the mouse. We present conditional genetic lineage-tracing data from mice supporting the hypothesis that progenitors expressing the glial marker *proteolipid protein 1*, presumably located in adjacent ganglia/nerves, also contribute to glomus cells. Finally, we resolve a paradox for the ‘émigré’ hypothesis in the chicken - where the nearest ganglion to the carotid body is the nodose, in which the satellite glia are neural crest-derived, but the neurons are almost entirely placode-derived - by fate-mapping putative nodose neuronal 'émigrés' to the neural crest.

## Highlights

• Glomus cell precursors express the neuron-specific marker Elavl3/4 (HuC/D).
• Developing glomus cells express multiple ‘sympathoadrenal' genes.
• Glomus cell development requires *Hand2* and *Sox4/11*, but not *Ret* or *Tfap2b*.
• Multipotent progenitors with a glial phenotype contribute to glomus cells.
• Fate-mapping resolves a paradox for the ganglionic 'émigré' hypothesis in birds.

## 1 Introduction

**Fig. S1:**
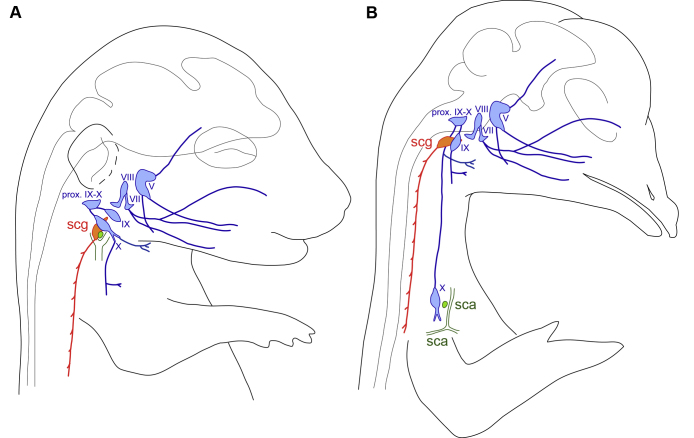
**Schematic illustrating the position of the carotid body relative to nearby ganglia and arteries.** (**A**) In the mouse, the nearest ganglion to the carotid body (light green) at the bifurcation of the carotid artery (dark green) is the superior cervical ganglion (scg, orange), medial to the carotid body. The nodose ganglion (distal ganglion of cranial nerve X, blue) and petrosal ganglion (distal ganglion of cranial nerve IX, blue) ganglia are also nearby. Schematic modified from Fig. 1 in McDonald and Mitchell (1975). (**B**) In the chicken, the carotid artery bifurcation (dark green), carotid body (light green) and nodose ganglion (distal ganglion of cranial nerve X, blue) have descended caudally into the elongated neck. The superior cervical ganglion (orange) and petrosal ganglion (distal ganglion of cranial nerve IX, blue) are found at the level of the jaws. Schematic modified from Fig. 2 in D'Amico-Martel and Noden (1983). Roman numerals indicate the cranial nerve associated with the indicated sensory ganglia (V, trigeminal; VII, facial; VIII, vestibuloacoustic; IX, glossopharyngeal; X, vagal). cca, common carotid artery; prox. proximal ganglion of cranial nerve; sca, subclavian artery; scg, superior cervical ganglion.

The carotid body is the primary site for monitoring arterial blood hypoxia (low O_2_ partial pressure) and hypercapnia (high CO_2_ partial pressure) in amniotes, triggering appropriate respiratory responses for homeostasis (Nurse and Piskuric, 2013, Nurse, 2014, López-Barneo et al., 2016). This small, highly vascularised chemosensory organ is entirely neural crest-derived, as shown by quail-chick grafting experiments in birds (Le Douarin et al., 1972, Pearse et al., 1973) and genetic lineage tracing in mice (Pardal et al., 2007). It develops at the bifurcation of the carotid artery (Fig. S1A,B), which derives from the third pharyngeal arch artery, whose wall (excepting endothelial cells) is derived from the neural crest (Le Lièvre and Le Douarin, 1975, Jiang et al., 2000b, Etchevers et al., 2001). In the mature carotid body, a dense capillary network surrounds groups of electrically excitable, neuroendocrine glomus (type I) cells and glial-like sustentacular (type II) cells. Sustentacular cells act as adult stem cells for glomus cell production upon exposure to chronic hypoxia, e.g. at high altitude (Pardal et al., 2007). Arterial blood hypoxia causes glomus cell membrane depolarisation and the release of neurotransmitters (including acetylcholine, ATP, the catecholamine dopamine, and the monoamine serotonin) onto glossopharyngeal carotid sinus nerve afferents in mammals (Nurse and Piskuric, 2013, Nurse, 2014, López-Barneo et al., 2016) (originating from the petrosal ganglion, i.e., the inferior ganglion of cranial nerve IX) and vagal afferents in birds, originating from the nodose ganglion (inferior ganglion of cranial nerve X) (Kameda, 2002). The carotid body also receives sympathetic innervation, from the superior cervical ganglion in mouse (Kameda et al., 2008) and from the 14th cervical sympathetic ganglion in the chicken (Kameda, 2002). Since glomus cells produce not only dopamine but also glial cell line-derived neurotrophic factor (GDNF), which promotes the survival of dopaminergic neurons (Erickson et al., 2001), the carotid body is a potential source of patient-specific stem cells for Parkinson's disease therapy (Espejo et al., 1998, Luquin et al., 1999, Mínguez-Castellanos et al., 2007, López-Barneo et al., 2009).

Intriguingly, the neural crest-derived chromaffin cells of the adrenal medulla (so named because they exhibit a "chromaffin" - chromium salt-staining - phenotype, attributed to the large cytoplasmic granules in which catecholaminergic neurotransmitters are stored; Pearse et al., 1973; Varndell et al., 1982) are also hypoxia-sensitive. Before mammalian adrenal chromaffin cells receive pre-ganglionic efferent innervation from neurons in the spinal cord, they respond to hypoxia by releasing catecholamines, as demonstrated in newborn or foetal calves, sheep, and rats (Comline and Silver, 1966, Cheung, 1989, Cheung, 1990, Seidler and Slotkin, 1985, Seidler and Slotkin, 1986, Thompson et al., 1997, Levitsky and López-Barneo, 2009). Similarly, neonatal rat adrenal chromaffin cells release catecholamines in response to hypercapnia (Muñoz-Cabello et al., 2005). Even the adult adrenal medulla retains some hypoxia-sensitive chromaffin cells (García-Fernández et al., 2007, Levitsky and López-Barneo, 2009) (see also López-Barneo et al., 2016). In both glomus cells and adrenal chromaffin cells, K^+^ channels are inhibited by hypoxia (reviewed by López-Barneo et al., 2016), while the “set point” at which glomus cells and adrenal chromaffin cells respond to hypoxia is regulated in both cell types by mutual antagonism between the transcription factors hypoxia-inducible factor 1-alpha (Hif1α/HIF1a) and hypoxia-inducible factor 2-alpha (Hif2α/HIF2a) (Yuan et al., 2013). Stabilising mutations in HIF2A, as well as mutations in at least 11 other genes, cause tumours in both the carotid body (head and neck paragangliomas, for which the carotid body is the most common site) and adrenal medulla (pheochromocytomas) (reviewed by Maher, 2014; Favier et al., 2015; Castro-Vega et al., 2016).

These common features and pathologies suggest that glomus cell and adrenal chromaffin cell development may involve similar genetic pathways, despite originating from markedly different axial levels of the neural crest: vagal for the carotid body, at the level of somites (s)1–7 (Le Douarin et al., 1972, Pearse et al., 1973); rostral to the hindlimb for adrenal chromaffin cells, at the level of s18–24 (Le Douarin and Teillet, 1971). The molecular mechanisms underlying the development of adrenal chromaffin cells have been extensively studied (reviewed by Huber, 2006, Huber, 2015; Huber et al., 2009; Kameda, 2014; Lumb and Schwarz, 2015) (also see Furlan et al., 2017). However, the molecular mechanisms underlying glomus cell development remain obscure, apart from a requirement, as in sympathetic neurons and adrenal chromaffin cells, for the key autonomic neural crest transcription factors Phox2b (Dauger et al., 2003, Huber et al., 2005) and Ascl1 (Mash1) (Huber et al., 2002, Kameda, 2005) (also see Kameda, 2014).

Recently, genetic lineage-tracing using tamoxifen-inducible Cre drivers that label peripheral glial cells - *Sox10*^*CreERT*2^ (Laranjeira et al., 2011) and *proteolipid protein 1*^*CreERT2*^ (*Plp1*^*CreERT2*^; Leone et al., 2003) - crossed with the *R26R*^*YFP*^ reporter line (Srinivas et al., 2001) revealed that multipotent progenitors with a glial phenotype ("Schwann cell precursors"), associated with the preganglionic sympathetic nerve fibres that innervate the adrenal medulla, make a significant contribution to the adrenal chromaffin cell population (Furlan et al., 2017). This is in addition to the segregation of chromaffin cell precursors at the dorsal aorta (see e.g. Saito et al., 2012). Glomus cell precursors have long been described, based on histological analysis, as ‘émigrés’ from neighbouring ganglia and/or nerves, both in a range of mammalian embryos including human (e.g. Kohn, 1900; Smith, 1924; Korkala and Hervonen, 1973) and in chicken embryos (Kameda, 1994, Kameda, 2002, Kameda et al., 1994). Analysis of various mutant mouse embryos has also suggested that glomus cell development requires the presence of both the adjacent superior cervical ganglion (Fig. S1A), which provides sympathetic innervation to the carotid body, and the afferent carotid sinus nerve (a branch of the glossopharyngeal nerve, originating from the petrosal ganglion) (Kameda, 2006, Kameda et al., 2008) (also see Kameda, 2014). These descriptive data raise the possibility that multipotent progenitors with a glial phenotype might contribute to glomus cells, as well as to adrenal chromaffin cells (Furlan et al., 2017).

Here, we investigate molecular and cellular aspects of glomus cell development in chicken and mouse, and report many striking similarities (but also some differences) with adrenal chromaffin cell development. We provide evidence supporting the hypothesis that progenitors with a glial phenotype contribute to glomus cells. Finally, we resolve a paradox for the neuronal ‘émigré’ hypothesis of glomus cell origins in the chicken, where the nearest ganglion to the carotid body is the nodose (Fig. S1B), whose neurons are almost entirely placode-derived, rather than neural crest-derived (Narayanan and Narayanan, 1980, D’Amico-Martel and Noden, 1983, Kious et al., 2002).

## 2 Materials and methods

### 2.1 Ethics statement

Experiments using chicken (*Gallus gallus domesticus*) embryos were conducted in accordance with the UK Animals (Scientific Procedures) Act 1986. Experiments involving crosses between *Wnt1-Cre* mice (Danielian et al., 1998) and *Hand2*^*flox/flox*^*;R26R*^*YFP*^ mice (Hendershot et al., 2008, Srinivas et al., 2001) were approved by the University of Toledo Health Sciences Campus Institutional Animal Care and Use Committee. Experiments involving the generation of *Wnt1-Cre*;*Sox4*^*flox/flox*^*;Sox11*^*flox/flox*^ embryos (Danielian et al., 1998, Bhattaram et al., 2010, Potzner et al., 2010) were conducted in accordance with German Animal Care laws and approved by the responsible governmental agency of Unterfranken. Experiments involving *Ret* knockout mice (Baudet et al., 2008) and *Plp1*^*CreERT2*^ mice (Leone et al., 2003) were conducted according to The Swedish Animal Agency's Provisions and Guidelines for Animal Experimentation recommendations and approved by the Ethical Committee on Animal Experiments (Stockholm North committee). Experiments involving *Tfap2b* knockout mice (Moser et al., 1997) were approved by the Vanderbilt University Institutional Animal Care and Use Committee.

### 2.2 Chicken and mouse embryos

Fertilised wild-type chicken eggs were obtained from commercial sources. Fertilised GFP-transgenic chicken eggs (McGrew et al., 2008) were obtained from the Roslin Institute Transgenic Chicken Facility (Edinburgh, UK), which is funded by Wellcome and the BBSRC. Embryos from the following mouse lines were obtained and genotyped as previously described: combination of the *Wnt1-Cre* transgene (Danielian et al., 1998) with *Hand2*^*flox/flox*^*;R26R*^*YFP*^ alleles (Hendershot et al., 2008, Srinivas et al., 2001) or *Sox4*^*flox/flox*^*;Sox11*^*flox/flox*^ alleles (Bhattaram et al., 2010, Potzner et al., 2010); *Ret* knockout mice (Baudet et al., 2008); *Tfap2b* knockout mice (Moser et al., 1997) and *Plp1*^*CreERT2*^ mice (Leone et al., 2003). Lineage-tracing experiments using the *Plp1*^*CreERT2*^ line were performed using heterozygotes for both the *Plp1*^*CreERT2*^ and *R26R*^*YFP*^ reporter lines. Tamoxifen (Sigma, T5648) was dissolved in corn oil (Sigma, C8267) and injected intraperitoneally into pregnant females at 0.1 mg/g body weight. Embryos were immersion-fixed overnight in 4% paraformaldehyde in phosphate-buffered saline at 4 °C.

### 2.3 *In situ* hybridisation and immunostaining on sections

Chicken embryos were incubated in a humidified atmosphere at 38 °C to the desired stage, fixed in modified Carnoy's solution (6 volumes ethanol, 3 volumes 37% formaldehyde, 1 volume glacial acetic acid), embedded for wax sectioning and sectioned at 6 µm. Mouse embryos were sucrose-protected before being embedded in O.C.T. (Tissue Tek), flash-frozen in isopentane on dry ice and cryosectioned at 10–15 µm. Sections were processed for *in situ* hybridisation and immunostaining as described previously (Moser et al., 1997, Miller et al., 2017). For all genetically modified mouse embryos, we analysed serial sections encompassing the entire region at the level of the superior cervical ganglion and carotid artery bifurcation to ensure the carotid body phenotype was accurately described.

Some clones used for making riboprobes were gifts, as follows: chicken *Gata2* and mouse *Gata3* from M. Salminen, University of Helsinki, Finland; *Hif1a* and *Hif2a* (*Epas1*) from G. Sheng, RIKEN Center for Developmental Biology, Kobe, Japan; mouse *Hand2*, *Phox2b* and *TH* from H. Rohrer, Max Planck Institute for Brain Research, Frankfurt, Germany; chicken *Phox2b* from J.-F. Brunet, IBENS, Paris, France; chicken *Ret* and *Sox10* from M. Bronner, Caltech, Pasadena, CA, USA; chicken *Sox11* from P. Scotting, University of Nottingham, Nottingham, UK.

Primary antibodies were: anti-GFP (1:500 rabbit, A-6455 Invitrogen; 1:500 goat, ab6662 Abcam); anti-Elavl3/4 (anti-HuC/D: 1:500 mouse IgG2b, A-21271 Invitrogen); anti-S100 (1:100 rabbit, Z0311 Dako); anti-serotonin (1:250 rabbit, S5545 Sigma-Aldrich; 1:2000 rabbit, 20080 Immunostar); anti-Sox4 (1:1500 guinea-pig; Hoser et al., 2008); anti-Sox11 (1:500 guinea-pig; Hoser et al., 2008); anti-Tubb3 (neuronal β-III tubulin: 1:500 mouse IgG2a, clone TUJ1, MMS-435P Covance BioLegend). AlexaFluor-conjugated secondary antibodies were obtained from Invitrogen.

### 2.4 *In ovo* grafting and electroporation

Fertilised wild-type and GFP-transgenic eggs were incubated in a humidified atmosphere at 38 °C for approximately 1.5 days to reach 6–11 somites and embryos visualised as previously described (Dude et al., 2009), using filtered phosphate-buffered saline instead of Ringer's solution. To label premigratory vagal neural crest cells, neural fold between the level of the otic vesicle and the caudal end of (1) somite 1 (s1) (unilaterally), (2) s6 (unilaterally) or (3) s4 (bilaterally) was grafted isotopically from GFP-transgenic donors to wild-type hosts using a pulled glass needle. For nodose placode labelling, the ectoderm overlying s1–2 (D’Amico-Martel and Noden, 1983, Baker and Bronner-Fraser, 2000) was similarly grafted unilaterally. Donor and host embryos were not always precisely stage-matched. Alternatively, we co-electroporated 6–12 somite-stage prospective nodose placode ectoderm with a Tol2-transposable GFP construct (*pT2K-CAGGS-EGFP*; Sato et al., 2007) and a Tol2 transposase construct (*pCAGGS-T2TP*; Sato et al., 2007) (gifts of Y. Takahashi, Kyoto University, Japan) at 5 μg/μl (2% Fast Green), using a TSS20 Ovodyne electroporator (Intracel) to apply four or five 50-ms 4 V pulses at 15-s intervals.

### 2.5 Image capture and analysis

Images of chicken and mouse embryo sections were captured using a Zeiss AxioSkop 2 MOT microscope fitted with a QImaging Retiga 2000R camera and an RGB pancake (Qimaging) and QCapture Pro 6.0 software, except *Plp1*^*CreERT2/+*^*;R26R*^*YFP/+*^ mouse embryos, for which images were captured on a Zeiss LSM 780 confocal microscope with Zeiss ZEN 2011 (black edition) software.

Counting of TH-positive adrenal chromaffin cells on mouse embryo sections was performed by using the “Threshold” function in ImageJ (NIH) to segment the area of TH expression, converting the segmented area into a region, applying this selection to the DAPI channel and using the “Find Maxima” function to count the nuclei within the selected region (with manually adjusted noise tolerance).

To determine the extent of overlap between serotonin and YFP reporter expression in the carotid bodies of *Plp1*^*CreERT2/+*^*;R26R*^*YFP/+*^ mouse embryos, high-resolution confocal image z-stacks were captured from 14 µm sections and the area of serotonin and YFP signal determined in ImageJ using a custom-written script (provided in the Supplementary information file). The script calculated the number of positive pixels in each channel in each ~ 0.8 µm slice of each stack (6–20 slices per stack) using the “Threshold” function with a static threshold value followed by the “getHistogram” function. For each ~ 0.8 µm slice, the separate channel windows were merged into a composite, and the number of pixels that overlapped in the thresholded channels was calculated using the “imageCalculator” and “getHistogram” functions. The number of thresholded pixels per ~ 0.8 µm slice in each category (i.e., thresholded as serotonin^+^, as YFP^+^, and as overlapping serotonin^+^YFP^+^) was summed to generate a total per stack for each category (the output of the ImageJ script). Microsoft Excel was used to sum the number of thresholded pixels in each category per carotid body (1–3 stacks per carotid body), and to calculate the percentage overlap (i.e., the percentage of thresholded serotonin^+^ pixels that were also thresholded as YFP^+^).

### 2.6 Statistical analysis

Data analysis and statistical tests were performed using Microsoft Excel and GraphPad Prism 7 software.

### 2.7 Code availability

The custom-written ImageJ script is provided in the Supplementary Information file.

## 3 Results

The sensitivity of neonatal adrenal chromaffin cells to both hypoxia (Comline and Silver, 1966, Cheung, 1989, Cheung, 1990, Seidler and Slotkin, 1985, Seidler and Slotkin, 1986, Thompson et al., 1997, García-Fernández et al., 2007, Levitsky and López-Barneo, 2009, López-Barneo et al., 2016) and hypercapnia (Muñoz-Cabello et al., 2005) led us to test the hypothesis that similar mechanisms underlie glomus cell and adrenal chromaffin cell development.

### 3.1 Developing chicken glomus cells express multiple ‘sympathoadrenal’ genes and the neuron-specific marker Elavl3/Elavl4 (HuC/D)

**Fig. 1:**
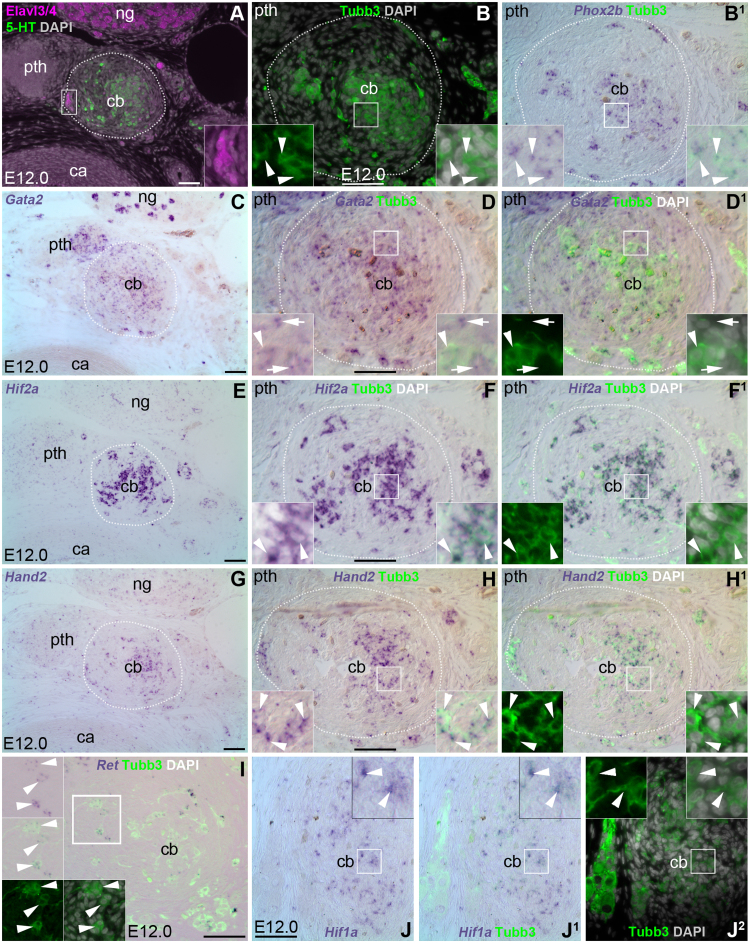
**Tubb3**^**+**^**glomus cells within the chicken carotid body at E12.0 express multiple ‘sympathoadrenal’ genes.** All panels show transverse sections in order from a series cut through the same E12.0 chicken embryo at the level of the carotid body and nodose ganglion. Dotted lines indicate the estimated periphery of the carotid body. Insets are magnified from the regions indicated by boxes. At least two embryos were analysed per gene at E12.0–E13.5 for *Phox2b*, *Gata2*, *Hif2a* and *Ret*; expression of *Hand2* and *Hif1a* was examined on serial sections of the same E12.0 embryo as the other genes. (**A,B**) At E12.0, the carotid body is a rounded structure separate from the carotid artery wall, adjacent to the parathyroid gland and nodose ganglion. The carotid body core is densely populated by serotonin-expressing (A, green), Tubb3^+^ (B, green) glomus cells. A few Elavl3/4-expressing neurons (A, magenta; shown at higher-power in inset) are present at the carotid body periphery, as well as in the adjacent nodose ganglion. (**B**^**1**^**-J**^**2**^) Tubb3^+^ glomus cells (green) in the core of the carotid body at E12.0 express *Phox2b* (B,B^1^), *Gata2* (C-D^1^; also expressed by nearby Tubb3-negative cells, presumably sustentacular cells: examples indicated by arrows in inset in D,D^1^; the arrowhead highlights a *Gata2*-positive glomus cell), *Hif2a* (E-F^1^), *Hand2* (G-H^1^), *Ret* (I; expression is weak but detectable in most or all glomus cells) and *Hif1a* (J-J^2^, showing the contralateral carotid body to the other panels). 5-HT, serotonin; ca, carotid artery; cb, carotid body; ng, nodose ganglion; pth, parathyroid gland. Scale-bar: 50 µm.

As a first step, we explored ‘sympathoadrenal’ gene expression (see Huber, 2006, Huber, 2015; Huber et al., 2009; Kameda, 2014; Lumb and Schwarz, 2015) during chicken carotid body development. At embryonic day (E)12.0–E13.5, the carotid body can easily be identified as a rounded, glandular structure, separate from the carotid artery wall, adjacent to the nodose (inferior vagal) ganglion and parathyroid gland (Fig. 1A). As expected from previous work (Kameda et al., 1994), serotonergic, Tubb3 (neuronal βIII-tubulin)-immunoreactive glomus cells were found in the core (Fig. 1A,B). Co-immunostaining for the neuron-specific Elav-like RNA binding proteins Elavl3/Elavl4 (HuC/D) (Okano and Darnell, 1997, Pascale et al., 2008) (hereafter Elavl3/4) labelled not only the neurons in the adjacent nodose ganglion, but also some neurons at the carotid body periphery (Fig. 1A). In addition to *Phox2b* (Fig. 1B^1^), whose expression was expected given its requirement for mouse glomus cell differentiation (Dauger et al., 2003), the Tubb3-positive glomus cells in the carotid body core expressed several previously unreported ‘sympathoadrenal’ genes (Fig. 1C-J^2^): *Gata2* (also expressed in adjacent Tubb3-negative cells, presumably sustentacular cells, as well as in the parathyroid gland and a subset of nodose neurons), *Hif2a* (*Epas1*), *Hand2*, *Ret* and *Hif1a*. (Although in the mouse, *Gata3* is required for sympathetic neuron and adrenal chromaffin cell differentiation and survival, in the chicken, *Gata2*, not *Gata3*, is expressed during sympathetic ganglion development; Lim et al., 2000; Tsarovina et al., 2004; Moriguchi et al., 2006; Tsarovina et al., 2010.)

**Fig. 2:**
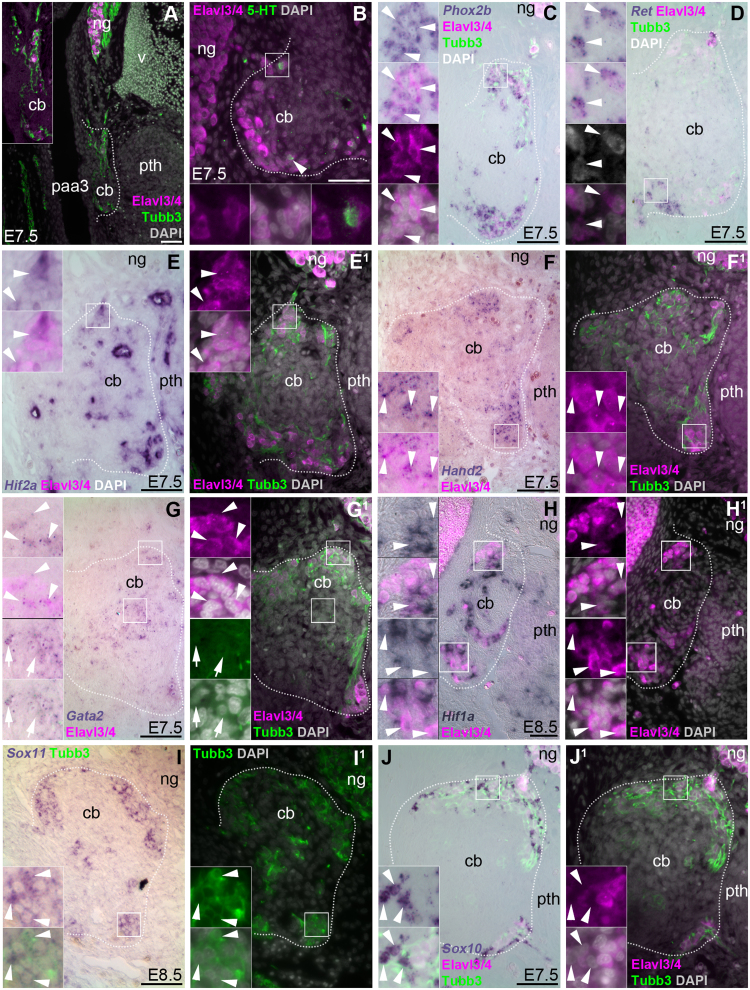
**Tubb3**^**+**^**cells at the periphery of the chicken carotid body at E7.5–8.5 express the neuron-specific RNA-binding proteins Elavl3/Elavl4 (HuC/D) and multiple ‘sympathoadrenal’ genes.** All panels show transverse sections of chicken embryos at E7.5–E8.5 at the level of the carotid body and nodose ganglion. Dotted lines indicate the estimated periphery of the carotid body. Insets are magnified from the regions indicated by boxes. Panels A and C-H^1^ show sections from the same E7.5 embryo; panel B shows a section from a different E7.5 embryo. At least 3 embryos were analysed per gene at E7.5–E9.5 (some of the embryos analysed for expression of the positive control *Phox2b* had received unilateral grafts of hindbrain neural folds to label neural crest-derived cells; the data shown are from an unmanipulated embryo). (**A**) At E7.5, the developing carotid body can be identified as a bulge from the wall of the third pharyngeal arch artery (future carotid artery), between the nodose ganglion and the parathyroid gland, surrounded by Tubb3^+^ neurites (green). Tubb3^+^ cells (green) at the carotid body periphery (shown at higher-power in the inset) are immunoreactive for the neuron-specific RNA-binding proteins Elavl3/Elavl4^+^ (Elavl3/4; magenta). (**B**) At E7.5, a few of the Elavl3/4^+^ neurons at the carotid body periphery express serotonin (5-HT; green, arrowheads). (**C-J**^**1**^) Neurons at the carotid body periphery express *Phox2b* (C), *Ret* (D), *Hif2a* (E,E^1^; expression only seen in a subset of neurons, e.g. compare *Hif2a* and Elavl3/4 distribution in main panels E,E^1^; intense signal marks endothelial cells), *Hand2* (F,F^1^), *Gata2* (G,G^1^; also expressed by non-neuronal cells in the carotid body core - arrows in second [bottom] inset highlight examples), *Hif1a* (H,H^1^; expression only seen in a subset of neurons, e.g. compare *Hif1a* and Elavl3/4 distribution in first [top] inset in panels H,H^1^) and *Sox11* (I,I^1^). The neural crest-derived peripheral neuronal precursor/glial marker *Sox10* was also expressed in non-neuronal cells associated with the neurons at the periphery of the carotid body (Fig. 2J,J^1^). [If shown in serial section order, this section would be located between panels F (*Hand2*) and G (*Gata2*).] 5-HT, serotonin; cb, carotid body; ng, nodose ganglion; paa3, third pharyngeal arch artery; pth, parathyroid gland; v, vein. Scale-bar: 50 µm.

We also looked earlier in carotid body development, focusing on the third pharyngeal arch artery (future carotid artery) near the nodose ganglion, where the primordia of the carotid body and adjacent parathyroid gland can be detected histologically from E6 (stage 29; Hamburger and Hamilton, 1951; Kameda et al., 1994). Tubb3-immunoreactive cells were reported as encircling the developing carotid body at E8 but increasingly located within the carotid body parenchyma from E9–10, with the first immunoreactivity at E10 for both serotonin and the catecholamine biosynthesis enzyme tyrosine hydroxylase (TH) (Kameda et al., 1994) (also see Kameda, 1990; Kameda et al., 1990). At E7.5–E9.5, the carotid body was identifiable as a bulge from the carotid artery wall, lying near the nodose ganglion and the parathyroid gland (Fig. 2A). The carotid body primordium was surrounded by Tubb3-positive neurites and Tubb3-positive cells, which were also immunoreactive for Elavl3/4 (Fig. 2A inset; Fig. 2B). A few of these Elavl3/4-positive neurons already expressed serotonin, i.e., were starting to differentiate as glomus cells (Fig. 2B). As well as *Phox2b* (Fig. 2C), the neurons at the carotid body periphery expressed multiple previously unreported ‘sympathoadrenal’ genes at E7.5-E9.5 (Fig. 2D-I^1^): *Ret*, *Hif2a* (*Epas1*), *Hand2*, *Gata2* (also expressed in many non-neuronal cells in the core of the developing carotid body), *Hif1a* and *Sox11. Hif2a* and *Hif1a* (both of which are also expressed in endothelial cells) were expressed in only a subset of neurons (Fig. 2E,E^1^,H,H^1^). The neural crest-derived peripheral neuronal precursor/glial marker *Sox10* (Britsch et al., 2001) was also expressed in non-neuronal cells closely associated with the neurons at the periphery of the carotid body (Fig. 2J,J^1^).

Overall, these data suggest that glomus cell development is likely to involve many of the same genes as adrenal chromaffin cell development. Furthermore, in addition to expressing multiple 'sympathoadrenal' genes and Tubb3, glomus cell precursors are immunoreactive for the neuron-specific RNA-binding proteins Elavl3/4, whose expression seems to be down-regulated (although Tubb3 is maintained) as maturing glomus cells up-regulate serotonin and move into the carotid body core.

### 3.2 Mouse glomus cell development requires *Hand2* and *Sox4/Sox11*, but not *Ret* or *Tfap2b*

**Fig. 3:**
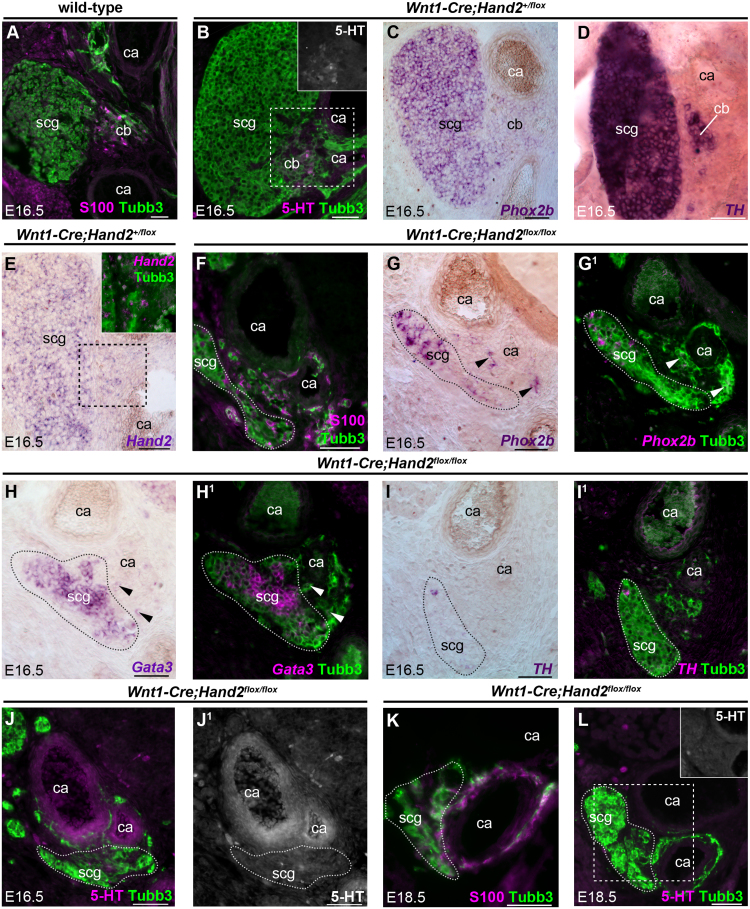
***Hand2*****is required in the neural crest for mouse glomus cell differentiation.** All panels show transverse sections of mouse embryos at E16.5 (A-J^1^) or E18.5 (K,L). Dotted lines indicate the estimated periphery of the carotid body or the superior cervical ganglion. (**A-E**) At E16.5, the carotid body can be identified at the carotid artery bifurcation, adjacent to the superior cervical ganglion, marked by S100 (A), serotonin (B, magnified in inset), *Phox2b* (C; stronger in the superior cervical ganglion than in the carotid body), *TH* (D) and *Hand2* (E; stronger in the superior cervical ganglion than in the carotid body). Panels B-E are sections from a series through a *Wnt1-Cre;Hand2*^*+/flox*^ embryo; panel A is from an unrelated wild-type embryo. (**F-J**^**1**^) Serial sections from an E16.5 *Wnt1-Cre;Hand2*^*flox/flox*^ mouse embryo (n = 5 from 1 litter), showing that after *Hand2* deletion in the neural crest lineage, the carotid body is absent and the superior cervical ganglion greatly reduced. S100 is still expressed at the carotid artery bifurcation, but scattered throughout the mesenchyme (F). Cells expressing *Phox2b* (G,G^1^) and *Gata3* (H,H^1^) are found inside the remnant superior cervical ganglion and scattered near the carotid artery walls (arrowheads). *TH* expression is greatly reduced in the superior cervical ganglion and absent at the carotid artery bifurcation (I,I^1^). Serotonin is absent at the carotid artery bifurcation (J,J^1^) (faint signal in blood vessel walls is autofluorescence; compare with blood cells). (**K,L**) Serial sections from an E18.5 *Wnt1-Cre;Hand2*^*flox/flox*^ mouse embryo (n = 5 from 3 litters): the carotid body remains absent and the superior cervical ganglion greatly reduced. S100 is still expressed in the mesenchyme surrounding the carotid artery wall (K), but serotonin is absent (L, magnified in inset). 5-HT, serotonin; ca, carotid artery; cb, carotid body; scg, superior cervical ganglion. Scale-bar: 50 µm.

**Fig. 4:**
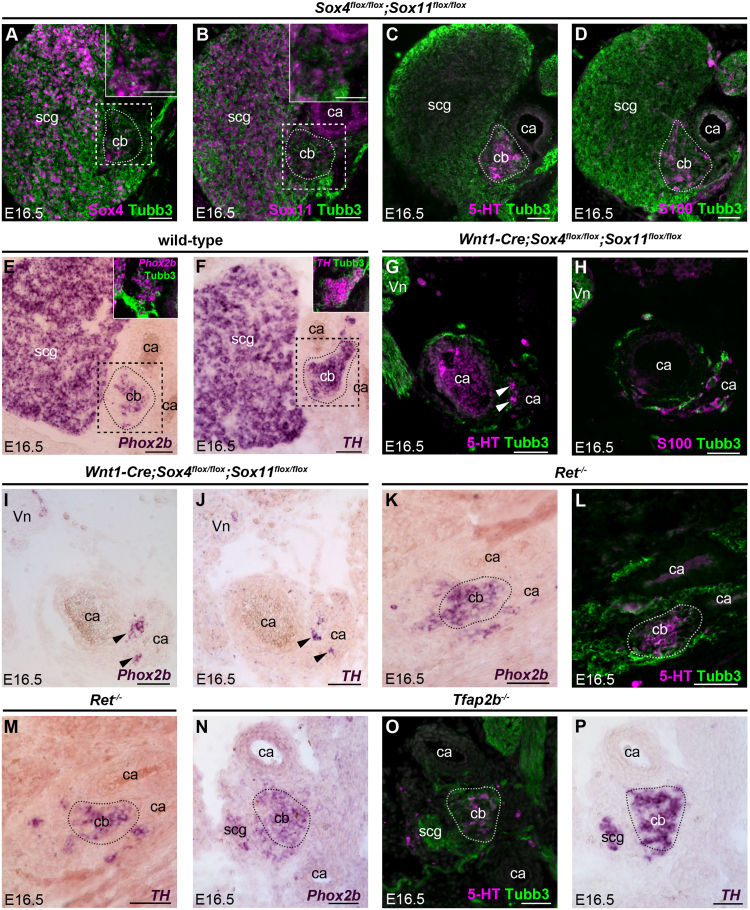
**Mouse glomus cells are depleted after neural crest-specific deletion of*****Sox4*****and*****Sox11*****, but unaffected by the loss of*****Ret*****or*****Tfap2b*****.** All panels show transverse sections of E16.5 mouse embryos. (**A-F**) The carotid body normally expresses both Sox4 (A) and Sox11 (B), as well as serotonin (C), S100 (D), *Phox2b* (E) and *TH* (F). Dashed boxes indicate (A,B) the region magnified in the insets, and (E,F) the overlap of gene expression (converted to magenta) with Tubb3 (green). Panels A-D are serial sections from a *Sox4*^*flox/flox*^*;Sox11*^*flox/flox*^ embryo; panels E and F are serial sections from an unrelated wild-type embryo. (**G-J**) Serial sections of a *Wnt1-Cre;Sox4*^*flox/flox*^*;Sox11*^*flox/flox*^ mouse embryo (n = 3 from 2 litters). After deletion of both *Sox4* and *Sox11* in the neural crest lineage, the superior cervical ganglion is residual/absent and the carotid body is highly reduced. Few serotonin-expressing glomus cells are present at the carotid artery bifurcation (arrowheads in G), although S100 is present diffusely in the mesenchyme surrounding the carotid arteries. The remnant glomus cells express *Phox2b* and *TH* (arrowheads in I,J). (Fig. S2 shows disruption of the organisation, but not the number, of adrenal medullary chromaffin cells.) (**K-M**) Serial sections of a *Ret*^-/-^ embryo (n = 3 from 3 litters). Although the superior cervical ganglion is small and aberrantly positioned (hence not seen in these sections; see Fig. S3), the carotid body develops normally at the carotid artery bifurcation, as indicated by *Phox2b* (K), serotonin (L) and *TH* (M). (**N-P**) Serial sections of a *Tfap2b*^-/-^ embryo (n = 5 from 3 litters). Although the superior cervical ganglion is highly reduced, the carotid body develops normally at the carotid artery bifurcation, as indicated by *Phox2b* (N), serotonin (O) and *TH* (P). Dotted lines in panels K-P indicate the estimated periphery of the carotid body. 5-HT, serotonin; ca, carotid artery; cb, carotid body; scg, superior cervical ganglion; vn, vagal nerve. Scale-bar: 50 µm.

In order to test the roles of some of these genes in glomus cell development, we turned to the mouse. At E16.5, the carotid body could be identified at the carotid artery bifurcation by double-immunostaining for Tubb3 and the sustentacular cell marker S100 or serotonin (Fig. 3A,B; see also Fig. 4C,D), or *in situ* hybridisation for *Phox2b* or *TH* (Fig. 3C,D; see also Fig. 4E,F). The carotid body was adjacent to (and had a Tubb3^+^ connection to) the superior cervical ganglion: the largest, most rostral sympathetic ganglion (see Fig. S1A). For all mutant mouse embryos, we analysed serial sections encompassing the entire region at the level of the superior cervical ganglion and carotid artery bifurcation, to ensure accurate description of the carotid body phenotype.

Consistent with our chicken data, the embryonic mouse carotid body expressed *Hand2* (Fig. 3E). To test the hypothesis that *Hand2* is required for glomus cell development, we analysed carotid body development in *Wnt1-Cre*;*Hand2*^*flox/flox*^ embryos (Hendershot et al., 2008, Srinivas et al., 2001), in which *Hand2* is specifically deleted in the neural crest lineage (Danielian et al., 1998, Hendershot et al., 2008). At E16.5 (n = 5 from 1 litter), the superior cervical ganglion was reduced and irregularly shaped (Fig. 3F-J^1^). Although S100^+^ cells associated with Tubb3^+^ cells were scattered near the carotid artery walls, there was no obvious carotid body condensation (Fig. 3F). *Phox2b* and *Gata3* were both expressed in the superior cervical ganglion remnant (Fig. 3G-J^1^), but only scattered cells near the carotid artery wall expressed these markers (arrowheads in Fig. 3G-H^1^): the *Phox2b*-positive cells, at least, may represent immature glomus cells. Few superior cervical ganglion cells expressed *TH* and there was no *TH* or serotonin expression near the carotid artery wall at E16.5 (Fig. 3I-J^1^) or at E18.5 (Fig. 3K,L) (n = 5 from 3 litters), indicating that carotid body maturation was not simply delayed. These results suggest that *Hand2* is required in the neural crest for glomus cell specification and maturation.

**Fig. S2:**
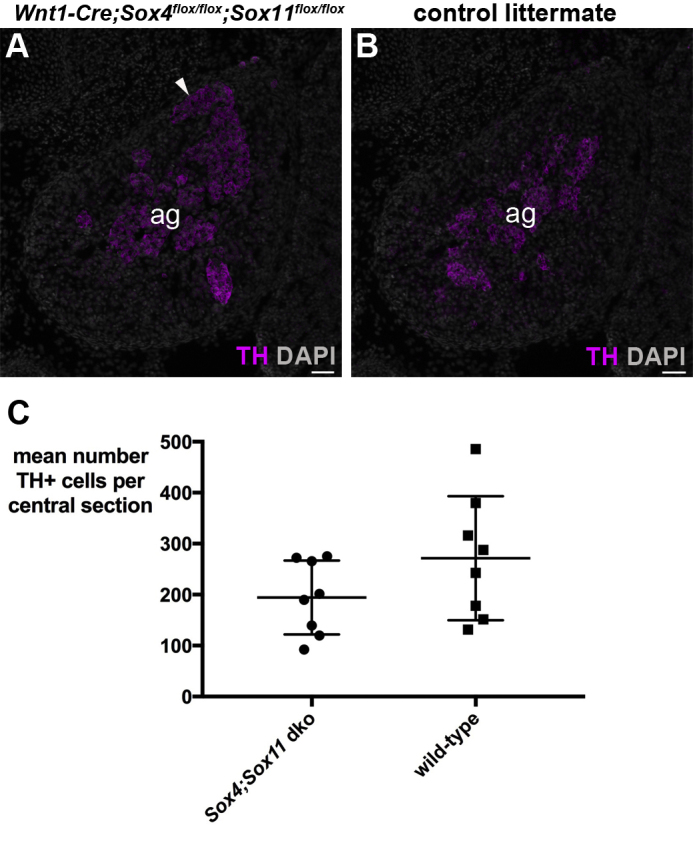
**Deletion of*****Sox4*****and*****Sox11*****in the neural crest disrupts the organisation of adrenal chromaffin cells, but has no significant effect on their numbers**. (**A,B**) Transverse sections at the level of the adrenal gland in E16.5 mouse embryos. Scale-bar: 50 µm. (A) After deletion of both *Sox4* and *Sox11* in the neural crest lineage (*Wnt1-Cre*;*Sox4*^*flox/flox*^*;Sox11*^*flox/flox*^ mice), TH^+^ adrenal chromaffin cells are disorganised, with some found in the cortex (arrowhead). (B) In control littermates, TH^+^ adrenal chromaffin cells are confined to the medulla. (**C**) Scatter-plot showing the mean number of TH^+^ adrenal chromaffin cells counted per central section of the adrenal gland at E16.5 (at least 3 sections counted through the central region of each adrenal gland across 4 embryos for each genotype). Bars indicate the overall mean and standard deviation: 194.5 ± 72.5 for *Wnt1-Cre*;*Sox4*^*flox/flox*^*;Sox11*^*flox/flox*^ double knockout (dko) mice (minimum 92.2; maximum 275.3) versus 271.5 ± 121.6 for control littermates (minimum 131.3; maximum 485.8). The overall means are not significantly different (unpaired 2-tailed Student’s *t*-test; p = 0.15, t = 1.539, 14 degrees of freedom). Each dataset passed the D’Agostino and Pearson normality test (alpha = 0.05; mutant dataset: K2 =2.00, p = 0.37; wild-type dataset: K2 = 0.70, p = 0.70). The F test shows that the variances are not significantly different (p = 0.20). dko, double knockout.

The SoxC transcription factor genes *Sox11* (which we found to be expressed in developing chicken glomus cells) and *Sox4* are respectively required for the early proliferation and later survival of TH-expressing cells in sympathetic ganglia (Potzner et al., 2010). At E16.5, Sox4 and Sox11 were both expressed in the superior cervical ganglion and adjacent carotid body (Fig. 4A,B), identifiable by serotonin, S100, *Phox2b* or *TH* expression (Fig. 4C-F). We analysed carotid body development in *Wnt1-Cre*;*Sox4*^*flox/flox*^*;Sox11*^*flox/flox*^ embryos (Danielian et al., 1998, Potzner et al., 2010), which have hypoplastic sympathetic ganglia owing to the lack of both Sox4 and Sox11 in the neural crest (Potzner et al., 2010). At E16.5 (n = 3 from 2 litters), the superior cervical ganglion was highly reduced or absent, as expected (Potzner et al., 2010). Only a few serotonergic glomus cells were present at the carotid artery bifurcation (Fig. 4G; compare with Fig. 4C). The sustentacular cell marker S100 was expressed in this region, but immunoreactivity seemed more diffuse than in wild-type (Fig. 4H; compare with Fig. 4D). The remnant glomus cells expressed *Phox2b* and *TH* (Fig. 4I,J; compare with Fig. 4E,F), suggesting they had differentiated normally. These results suggest that Sox4 and Sox11 are important for glomus cell precursor proliferation and/or survival, rather than glomus cell differentiation. The effect of deleting *Sox4* and *Sox11* on adrenal chromaffin cell formation had not previously been examined. In contrast to the dramatic reduction in glomus cell numbers, there was no significant impact on the number of TH-positive adrenal chromaffin cells at E16.5, although they were disorganised, with some being located in the cortex (Fig. S2A-C).

**Fig. S3:**
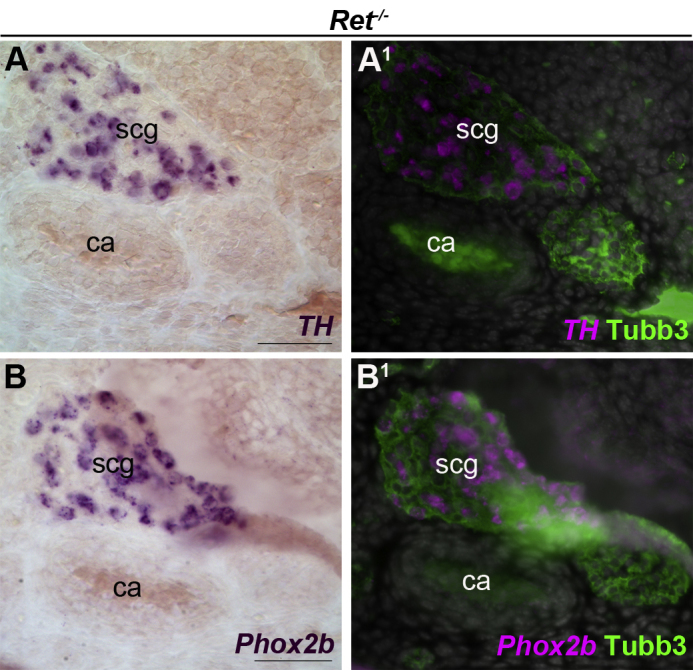
**The superior cervical ganglion is caudal to its wild-type position in*****Ret***^***-/-***^**mice.** All panels show transverse sections of the same E16.5 *Ret*^*-/-*^ mouse embryo shown in Fig. 4. (**A-B**^**1**^) The superior cervical ganglion is small and aberrantly positioned caudal to the carotid artery bifurcation; it can be identified adjacent to the carotid artery, in sections caudal to those shown in Fig. 4K-M, by the expression of *TH* (A, converted to magenta in A^1^) and *Phox2b* (B, converted to magenta in B^1^). ca, carotid artery; scg, superior cervical ganglion. Scale-bar: 50 µm.

In *Ret*-null embryos (Baudet et al., 2008) at E16.5, the superior cervical ganglion was small, aberrantly shaped and often positioned caudal to its normal location (Fig. S3A-B1). However, many glomus cells expressing *Phox2b*, serotonin and *TH* were present at the carotid artery bifurcation (Fig. 4K-M; n = 3 from 3 litters). Hence, *Ret* is not required for glomus cell aggregation or differentiation.

We also investigated carotid body development in mice lacking the transcription factor gene *Tfap2b*, which affects neurotransmitter expression and maturation of sympathetic neurons and adrenal chromaffin cells (Hong et al., 2008, Hong et al., 2011, Schmidt et al., 2011). In *Tfap2b*-null embryos (Moser et al., 1997) at E16.5 (n = 5 from 3 litters), the superior cervical ganglion was highly reduced (Fig. 4N-P), although the few sympathetic neurons present expressed *Phox2b* and *TH* (Fig. 4N,P). In contrast, many cells expressing *Phox2b*, serotonin and *TH* were present at the carotid artery bifurcation, suggesting normal glomus cell development in the absence of *Tfap2b* (Fig. 4N-P).

### 3.3 Conditional genetic lineage-tracing with a *Plp1^CreERT2^* driver line supports the hypothesis that multipotent progenitors with a glial phenotype contribute to glomus cells

The recent demonstration that multipotent progenitors with a glial phenotype make a significant contribution to adrenal chromaffin cells, based on conditional genetic lineage-tracing in the mouse (Furlan et al., 2017), raised the possibility that such progenitors - from adjacent ganglia/nerves - might also contribute to glomus cells. In the mouse, glomus cells receive afferent innervation from the carotid sinus nerve (a branch of the glossopharyngeal nerve, arising from the petrosal ganglion) and sympathetic innervation from the adjacent superior cervical ganglion (Hertzberg et al., 1994, Kameda et al., 2008). Indeed, carotid sinus afferents are already distributed around the third pharyngeal arch artery at E12.5, before the carotid body rudiment becomes evident at E13.0 (Hertzberg et al., 1994, Kameda et al., 2008).

In order to label peripheral glial cells and their descendants, we used the conditional *Plp1*^*CreERT2*^ driver line (Leone et al., 2003) employed in the adrenal chromaffin cell study (Furlan et al., 2017). *Plp1* encodes the main constituent of myelin in the central nervous system (Nave, 2010), but it is also expressed by Schwann cells along peripheral nerves and by satellite glia in peripheral ganglia (Kamholz et al., 1992, Jiang et al., 2000a). It was previously reported for *Plp1*^*CreERT2*^;*R26R*^*lacZ*^ double transgenic embryos that after tamoxifen-induced recombination at E12.5 (half a day before the carotid body rudiment can be detected at E13.0; Hertzberg et al., 1994; Kameda et al., 2008), beta-galactosidase-positive cells in the peripheral nervous system were distributed densely along nerves, and also in ganglia (Leone et al., 2003).

We crossed *Plp1*^*CreERT2/+*^ mice (Leone et al., 2003) with *R26R*^*YFP/+*^ reporter mice (Srinivas et al., 2001). Pregnant females were injected with tamoxifen at E12.5, or even later in development, at E15.5. Embryos were collected at E17.5 and 14 µm sections through the carotid body were immunostained for serotonin and YFP and imaged as high-resolution confocal z-stacks. It was difficult to identify individual serotonergic glomus cells in the confocal images, so we used a custom-written ImageJ script (Supplementary Information) to threshold serotonin^+^ pixels and YFP^+^ pixels in each ~0.8 µm slice of each image stack (6–20 slices per z-stack). For each ~0.8 µm slice, the script calculated the number of pixels thresholded as serotonin^+^, as YFP^+^, and (after merging the separate channel windows into a composite) as both serotonin^+^YFP^+^, and generated the total number of thresholded pixels for each category per z-stack. For each carotid body, 1–3 confocal image z-stacks were analysed, and the number of thresholded pixels per stack summed to give the total per carotid body for each category (i.e., serotonin^+^, YFP^+^, and overlapping serotonin^+^YFP^+^). The percentage overlap per carotid body was calculated as the percentage of all pixels thresholded as serotonin^+^ (i.e., belonging to glomus cells) that were also thresholded as YFP^+^ (i.e., belonging to the descendants of cells in which *Plp1*^*CreERT2*^ drove Cre expression and recombination following tamoxifen injection).

**Fig. 5:**
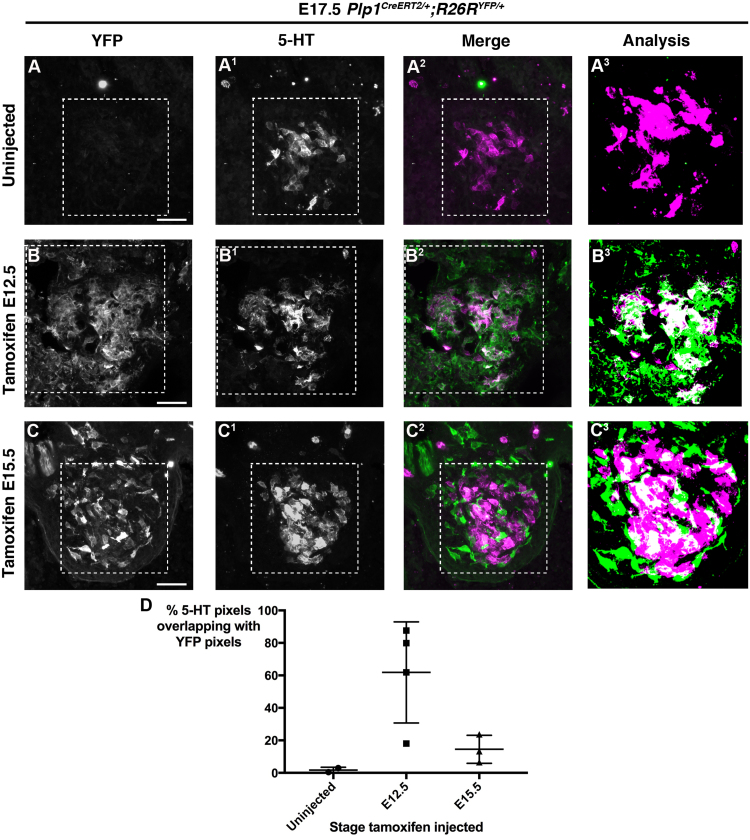
**Progenitors with a*****Plp1*****-expressing glial phenotype contribute to mouse glomus cells.** All panels show representative 3D-reconstructions created from confocal image z-stacks of 14 µm parasagittal sections through *Plp1*^*CreERT2/+*^*; R26R*^*YFP/+*^ mouse carotid bodies at E17.5, immunostained for serotonin and YFP. Pregnant females were either uninjected (**A-A**^**3**^) or injected with tamoxifen to induce recombination and reporter expression in glial cells at E12.5 (**B-B**^**3**^) or E15.5 (**C-C**^**3**^). The overlap of the serotonin and YFP signals (white pixels in analysis) was quantified in ImageJ, using a custom-written script (provided in Supplementary Information) that calculated for each ~0.8 µm slice of each z-stack the number of pixels thresholded as serotonin^+^ (5-HT; magenta), as YFP^+^ (green) and as both serotonin^+^ and YFP^+^, and summed the values per category for all the slices in each z-stack (6–20 slices per z-stack). For each carotid body, the values for all z-stacks analysed (1–3 per carotid body) were summed, and the overall overlap per carotid body calculated as the percentage of pixels thresholded as serotonin^+^ that were also thresholded as YFP^+^. Dashed boxes indicate the region that was analysed in each case. (**D**) Scatter-plot showing the percentage per carotid body of pixels thresholded as serotonin^+^ that were also thresholded as YFP^+^, for embryos from uninjected females, versus females injected with tamoxifen at E12.5 or at E15.5. Bars indicate mean ± s.d. (uninjected: mean 1.7 ± 1.8%, n = 2 carotid bodies from 2 embryos, 0.46% and 2.96% overlap, respectively; injected at E12.5: mean 61.9 ± 31.1%, n = 4 carotid bodies from 4 embryos; injected at E15.5: mean 14.5 ± 8.6%, n = 3 carotid bodies from 3 embryos). 5-HT, serotonin. Scale-bar: 40 µm.

In control embryos from uninjected mothers (Fig. 5A), the mean percentage± s.d. of pixels thresholded as serotonin^+^ that were also thresholded as YFP^+^ was minimal, as expected (1.7 ± 1.8%; n = 2 carotid bodies from 2 embryos: 0.46% and 2.96% overlap, respectively). In contrast, after tamoxifen-induced recombination at E12.5 (Fig. 5B), the mean percentage overlap ( ± s.d.) was 61.9 ± 31.1% (n = 4 carotid bodies from 4 embryos; min. 18.1%; max. 87.6%). When recombination was induced at E15.5 (Fig. 5C), the mean percentage overlap was considerably lower, at 14.5 ± 8.6% (n = 3 carotid bodies from 3 embryos; min. 6.5%; max. 23.7%), but was still greater than in the control uninjected embryos (as noted above, 1.7 ± 1.8%; n = 2 carotid bodies from 2 embryos). The data are presented as a scatter plot in Fig. 5D. Overall, these results support the hypothesis that in the mouse, peripheral progenitor cells with a *Plp1*-expressing (i.e., glial) phenotype, presumably located in nearby ganglia and/or nerves, contribute to glomus cells.

### 3.4 Resolving a paradox for the neuronal ‘émigré’ hypothesis of glomus cell origins in the chicken

Based on histological analysis and studies of mutant mouse embryos, glomus cells have long been suggested to develop from ‘émigrés’ from neighbouring ganglia (the superior cervical ganglion in mouse; Fig. S1A) and/or nerves, both in mammals (Kohn, 1900, Smith, 1924, Korkala and Hervonen, 1973, Kameda et al., 1994, Kameda et al., 2008, Kameda, 1994, Kameda, 2002, Kameda, 2006) and in the chicken (Kameda, 1994, Kameda, 2002, Kameda et al., 1994). In the chicken, however, the nearest ganglion to the carotid body is the nodose (inferior ganglion of the vagal nerve), which descends caudally into the neck (Narayanan and Narayanan, 1980, D’Amico-Martel and Noden, 1983) and lies between the carotid body and the cervical sympathetic trunk ganglia (Kameda, 2002) (Fig. S1B). While nodose satellite glia are neural crest-derived, nodose neurons (analysed in quail-chick chimeras ranging from E7–13) originate from the nodose placode (Narayanan and Narayanan, 1980, D’Amico-Martel and Noden, 1983). For the neuronal ‘émigré’ hypothesis of glomus cell origins to be correct, such ‘émigrés’ must be neural crest-derived, unlike the vast majority of nodose neurons. This possibility is supported by a previous report of a single neural crest-derived neuron in the nodose ganglion of one quail-chick neural fold chimera at E12.5 (D’Amico-Martel and Noden, 1983). Furthermore, a few neural crest-derived neurons have been described in both the petrosal and nodose ganglia of quail-chick neural fold chimeras at E3 (Kious et al., 2002). Taken together, these earlier reports suggest that some neural crest-derived neurons are initially present in the nodose ganglion, but have almost entirely disappeared by E12.5 - perhaps because they have emigrated to the carotid body.

**Fig. 6:**
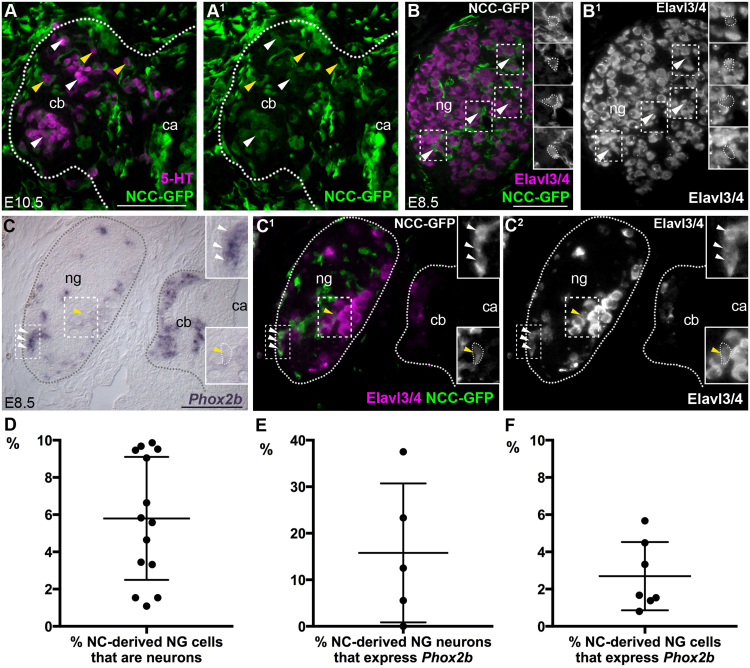
**Neural crest-derived neurons are present in the chicken nodose ganglion, some of which express the autonomic marker*****Phox2b***. Panels A-C^2^ show 6 µm transverse sections of chicken embryos fixed at the indicated stages after receiving vagal-level neural fold grafts from GFP-transgenic donors at E1.5. Table 1, Table 2 respectively show the numbers for vagal neural fold grafts and nodose ganglia analysed. (**A,A**^**1**^) Many serotonergic glomus cells (magenta) are GFP^+^, i.e., neural crest-derived (green; white arrowheads) (although not all are labelled; yellow arrowheads). (**B,B**^**1**^**)** In the nodose ganglion, a few neural crest-derived (GFP^+^) Elavl3/4^+^ neurons are present (white arrowheads, magnified in insets. Insets in B show GFP). **(C-C**^**2**^) Some neural crest-derived neurons in the nodose ganglion express *Phox2b* (white arrowheads, magnified in insets) (although not all are *Phox2b*^+^; yellow arrowhead, magnified in insets. Insets in C^1^ show GFP). (**D**) Scatter plot showing the percentage per nodose ganglion of neural crest-derived cells that are neurons at E5.5–E9.5 (bars indicate mean ± s.d.: 5.8 ± 3.3%; n = 14 ganglia from 14 embryos; between 130 and 1679 GFP^+^ cells counted per ganglion). (**E**) Scatter plot showing the percentage per nodose ganglion of neural crest-derived neurons that express *Phox2b* at E8.5–E9.5 (bars indicate mean ± s.d.: 15.8 ± 14.9%; n = 5 ganglia from 5 embryos; between 8 and 36 neural crest-derived neurons counted per ganglion). (**F**) Scatter plot showing the percentage per nodose ganglion of neural crest-derived cells that express *Phox2b* at E5.5−E9.5 (bars indicate mean ± s.d.: 2.7 ± 1.8%; n = 7 ganglia from 6 embryos [one E6.5 embryo had a significant contribution of GFP^+^ neural crest cells to both nodose ganglia, so each was analysed separately]; between 130 and 435 GFP^+^ cells counted per ganglion). 5-HT, serotonin; ca, carotid artery; cb, carotid body; NC, neural crest; NCC-GFP, GFP^+^ neural crest-derived cells; ng/NG, nodose ganglion. Scale-bar: 50 µm.

**Fig. S4:**
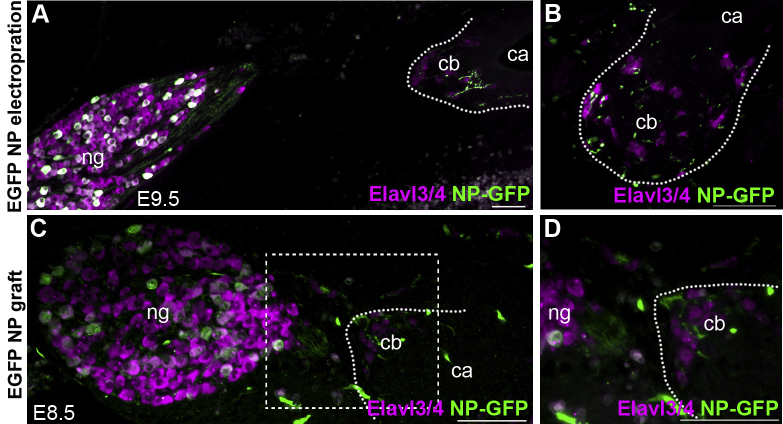
**Nodose placode-derived neurons do not contribute to the carotid body.** All panels show transverse sections of chicken embryos at the level of the nodose ganglion and carotid body (demarcated by dotted lines). (**A,B**) Sections of a wild-type embryo at E9.5 that had been electroporated at E1.5 with a Tol2-transposable construct encoding EGFP, allowing permanent labelling of nodose placode cells. Many GFP^+^ (green) Elavl3/4^+^ neurons (magenta) are present in the nodose ganglion (overlap shows as white), which extend GFP^+^ neurites into the carotid body. Elavl3/4^+^ -positive glomus cells in the carotid body do not express GFP, however. (**C,D**) Section of a wild-type embryo at E8.5 that had been grafted at E1.5 with prospective nodose placode ectoderm from a GFP-transgenic donor, enabling permanent labelling of nodose placode cells. GFP^+^ (green) Elavl3/4^+^ neurons (magenta) are present in the nodose ganglion (overlap shows as white) and GFP^+^ neurites project into the carotid body, but Elavl3/4^+^ glomus cells do not express GFP. The dashed box in C shows the region magnified in D. ca, carotid artery; cb, carotid body; ng, nodose ganglion; np, nodose placode; NP-GFP, GFP^+^ nodose placode-derived cells. Scale-bar: 50 µm.

In order to test whether putative neuronal ‘émigrés’ from the nodose ganglion are indeed neural crest-derived, rather than placode-derived like most nodose neurons (Narayanan and Narayanan, 1980, D’Amico-Martel and Noden, 1983, Kious et al., 2002), we first confirmed that the carotid body contains no nodose placode-derived cells (although it is innervated by nodose placode-derived neurites) by labelling prospective nodose placode ectoderm with GFP either by electroporation (n = 11; Fig. S4A,B) or grafting (n = 4; Fig. S4C,D). We then labelled premigratory vagal neural crest cells by bilaterally or unilaterally grafting vagal-level neural fold grafts from GFP-transgenic donor chick embryos (McGrew et al., 2008) to wild-type hosts. Such grafts labelled glomus cells, as expected (although the carotid body was never entirely GFP^+^ after such grafts in our hands) (Fig. 6A,A^1^; Table 1), and also a few Elavl3/4^+^ neurons in the nodose ganglion (Fig. 6B-C^2^). (We would expect most neural crest-derived cells in the nodose ganglion to be non-neuronal, since all the satellite glia are neural crest-derived; Narayanan and Narayanan, 1980; D’Amico-Martel and Noden, 1983). Cell counting revealed that after unilateral grafts, 6.2% of neural crest-derived cells in the nodose ganglion were neurons at E5.5-E9.5 (n = 376 out of 6067 GFP^+^ cells counted across 14 ganglia from 14 embryos). The mean per ganglion (± standard deviation, s.d.) was 5.8 ± 3.3% (n = 14; Fig. 6D; Table 2). Thus, as previously reported at E3 (Kious et al., 2002), the nodose ganglion contains some neural crest-derived neurons at E5.5-E9.5, when the carotid body is developing nearby.

**Table 1:** Breakdown of cell counting data for the mean percentage/carotid body of Elavl3/4^+^ or serotoninergic glomus cells that were GFP^+^ (neural crest-derived) after unilateral or bilateral vagal neural fold grafts from GFP-transgenic donors. E, embryonic day; n, number; NC, neural crest; Otic, otic placode level; S, somite level; s.d., standard deviation.

**Marker**	**Elavl3/4**			**Serotonin**			
**Graft type**	**Unilateral**			**Unilateral**		**Bilateral**	
**Region grafted**	**Otic to S3**	**Otic to S6**	**Total**	**Otic to S3**	**Otic to S6**	**Otic to S4**	**Total**
**Stage analysed**	E7.5–E9.5	E7.5–E9.5	E7.5–E9.5	E7.5–E9.5	E8.5–E9.5	E10.5	E7.5–E10.5
**Mean %NC-derived**	22.1	42.3	28.3	26.1	37.2	55.4	35.4
**s.d.**	10.7	33.6	21.2	9.7	16.2	7.7	15.9
**n carotid bodies**	9	4	13	7	3	5	15
**n embryos**	9	4	13	7	3	3	13
**n cells counted**	2034	472	2506	643	324	931	1898
**Minimum n cells**	44	22	22	10	23	197	10
**Maximum n cells**	511	251	511	328	187	512	512

**Table 2:** Breakdown of cell counting data for the mean percentage/nodose ganglion of neural crest-derived cells (GFP^+^) that expressed Elavl3/4 (i.e., that were neurons) or *Phox2b* after vagal neural fold grafts from GFP-transgenic donors. E, embryonic day; n, number; NC, neural crest; Otic, otic placode level; S, somite level; s.d., standard deviation.

**Marker**	**Elavl3/4**			***Phox2b***
**Graft type**	**Unilateral**			**Unilateral**
**Region grafted**	**Otic to S1**	**Otic to S6**	**Total**	**Otic to S1**^a^; **Otic to S6**
**Stage analysed**	E7.5–E9.5	E5.5–E9.5	E5.5–E9.5	E5.5–E9.5
**Mean % NC-derived**	9.6	4.3	5.8	2.7
**s.d.**	0.2	2.6	3.3	1.8
**n nodose ganglia**	4	10	14	7^b^
**n embryos**	4	10	14	6
**n cells counted**	2215	3852	6067	1875
**Minimum n cells**	310	130	130	130
**Maximum n cells**	782	1679	1679	435

a One E8.5 embryo.

b One unilaterally grafted E6.5 embryo had a large contribution of labelled neural crest cells to both nodose ganglia, so both were analysed.

Furthermore, 14.3% of the neural crest-derived neurons in the nodose ganglion expressed the autonomic marker *Phox2b* at E8.5–E9.5 (n = 13 out of 91 GFP^+^Elavl3/4^+^ cells counted across 5 ganglia from 5 embryos; Fig. 6C-C^2^), although the proportion per ganglion was variable (mean 15.8 ± 14.9%; n = 5; between 8 and 36 neural crest-derived neurons counted per ganglion; Fig. 6E; Table 2). (Nodose placode-derived neurons also express *Phox2b*; Pattyn et al., 1997.) The proportion of all neural crest-derived cells (as opposed to neurons) in the nodose ganglion that expressed *Phox2b* at E5.5–E9.5 was still 2.5% (n = 47 out of 1875 GFP^+^ cells counted across 7 ganglia from 6 embryos [one E6.5 embryo had a significant contribution of GFP^+^ neural crest cells to both nodose ganglia, so each was analysed separately]). The mean per ganglion was 2.7 ± 1.8% (n = 7; Fig. 6F; Table 2). Thus, at least some neural crest-derived cells in the nodose ganglion, including some neural crest-derived neurons, express the autonomic marker *Phox2b*.

**Fig. 7:**
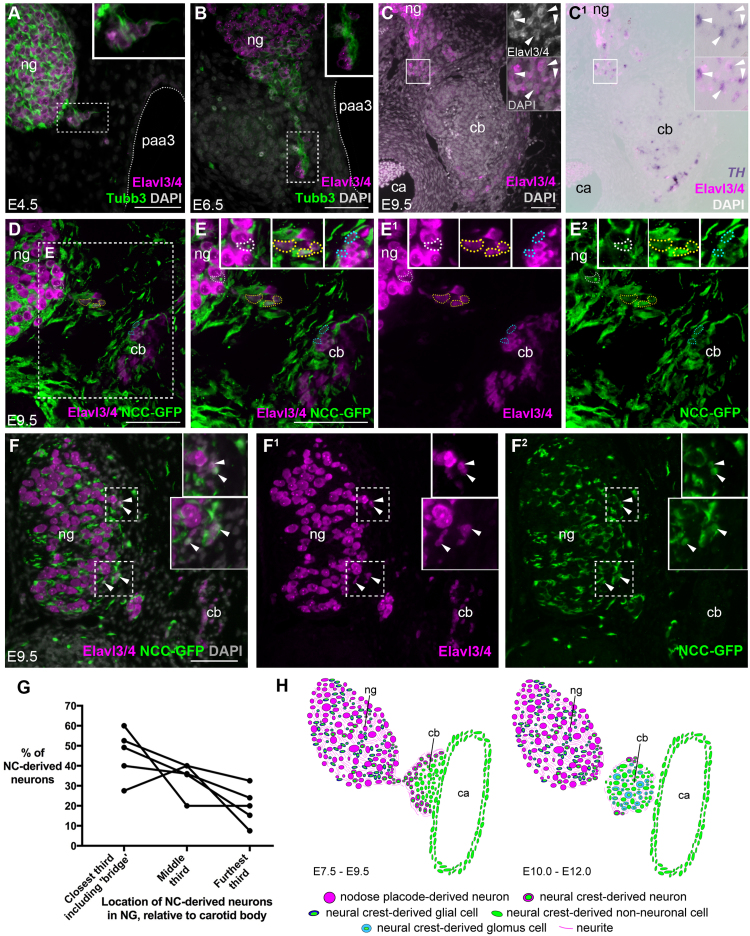
**Neural crest origin of the ‘bridge’ of neurons between the nodose ganglion and the developing chicken carotid body.** Panels A-F^2^ show 6 µm transverse sections of chicken embryos; insets are magnified from the regions indicated by boxes. Table 1 shows the number of embryos analysed at different stages after receiving vagal-level neural fold grafts from GFP-transgenic donors at E1.5. (**A,B**) At E4.5 (A) and E6.5 (B), Elavl3/4^+^ neurons and Tubb3^+^ neurites extend from the nodose ganglion towards the third pharyngeal arch artery wall (outlined with dotted line). (**C,C**^**1**^) At E9.5, the carotid body remains connected to the nodose ganglion by a bridge of Elavl3/4^+^ neurons, which express the catecholamine biosynthesis enzyme gene *TH*. (**D-E**^**2**^) An example from an E9.5 embryo that received a GFP-transgenic vagal neural fold graft at E1.5, with neural crest-derived (GFP^+^) Elavl3/4^+^ neurons (magenta) present at the edge of the nodose ganglion closest to the carotid body (white dotted outline), within the connecting bridge of Elavl3/4^+^ neurons (yellow dotted outline) and at the edge of the carotid body (blue dotted outline). (**F-F**^**2**^) An example from another E9.5 embryo that received a GFP-transgenic vagal neural fold graft at E1.5, with neural crest-derived (GFP^+^) Elavl3/4^+^ neurons (magenta) present on the side of the nodose ganglion closest to the carotid body (arrowheads). (**G**) Graph showing the proportion of neural crest-derived neurons located in different regions of one nodose ganglion and ‘bridge’ from each of 5 embryos at E8.5–E9.5 (between 10 and 59 neurons counted per ganglion). (**H**) Schematic summary of the cellular contributions to the developing chick carotid body. At E7.5–9.5, a bridge of neural crest-derived neurons projects from the nodose ganglion and surrounds the carotid body primordium, which forms a bulge from the carotid artery wall. By E10.0–12.0, the carotid body is separate from the carotid artery wall and the nodose ganglion. Neural crest-derived neuronal glomus cell precursors move from the periphery into the core of the carotid body, where they down-regulate neuronal markers and mature as catecholaminergic glomus cells. 5-HT, serotonin; ca, carotid artery; cb, carotid body; NC, neural crest; NCC-GFP, GFP^+^ neural crest-derived cells; ng/NG, nodose ganglion; paa3, third pharyngeal arch artery; pth, parathyroid. Scale-bar: 50 µm.

As previously reported (Kameda et al., 1994, Kameda, 1994, Kameda, 2002), “bridges” of neurons connect the nodose ganglion and the developing carotid body. In 5 out of 9 embryos that had received GFP-transgenic vagal neural fold grafts containing neural crest precursors, such “bridging” neurons were seen to be GFP^+^, i.e., neural crest-derived. As early as E4.5, a small cluster of Elavl3/4^+^ neurons and Tubb3^+^ neurites extended from the nodose ganglion towards the wall of the third pharyngeal arch artery (PAA3; future carotid artery) (Fig. 7A). At E6.5, a continuous “bridge” of neurons and neurites connected the nodose ganglion to neurons in the PAA3 wall (Fig. 7B). At E7.5–9.5, many neurons were found in the peripheral carotid body, connected to the nodose ganglion via a neuronal bridge (Fig. 7C-E^2^). Furthermore, the "bridge" neurons at E9.5 expressed the catecholamine biosynthesis gene *TH*, unlike placode-derived nodose ganglion neurons but like differentiating glomus cells in the adjacent carotid body (Fig. 7C,C^1^), consistent with their being glomus cell precursors. Neural crest-derived (GFP^+^) neurons were noted at all points in this neuronal bridge: at the edge of the nodose ganglion, in the bridge itself and at the periphery of the carotid body (Fig. 7D-E^2^ shows an example at E9.5). Moreover, consistent with the hypothesis of emigration, the location of the neural crest-derived neurons in the nodose ganglion and ‘bridge’ was biased towards the carotid body (Fig. 7F-G). This bias was sometimes apparent in sections (Fig. 7F-F^2^), but not always. However, quantification of the proportion of neural crest-derived neurons located in different regions of one nodose ganglion and ‘bridge’ from each of 5 embryos at E8.5–E9.5 (between 10 and 59 neurons counted per ganglion) showed a consistent bias towards the side closest to the carotid body (Fig. 7G). In contrast, out of 11 chicken embryos in which the prospective nodose placode ectoderm was labelled with GFP by electroporation, only one GFP^+^ placodal neuron was observed near the edge of the carotid body (data not shown). Fig. 7H shows a schematic summary model for the cellular contributions to the chicken carotid body.

Taken together, our data resolve the apparent paradox associated with the hypothesis that glomus cell precursors in the chicken are neurons emigrating from the adjacent nodose ganglion (Kameda et al., 1994, Kameda, 1994, Kameda, 2002), given that almost all nodose neurons are placode-derived (Narayanan and Narayanan, 1980, D’Amico-Martel and Noden, 1983, Kious et al., 2002). Instead, the putative neuronal 'émigrés' from the nodose ganglion are neural crest-derived (and express *TH*), further supporting the hypothesis that such émigrés are indeed glomus cell precursors.

## 4 Discussion

The neural crest-derived glomus cells of the carotid body are essential for ventilatory reflex responses to hypoxia and hypercapnia in arterial blood (Nurse and Piskuric, 2013, Nurse, 2014, López-Barneo et al., 2016), and are a potential source of patient-specific dopaminergic cells and/or sources of growth factors that support dopaminergic neurons, for Parkinson's disease therapy (Espejo et al., 1998, Luquin et al., 1999, Mínguez-Castellanos et al., 2007, López-Barneo et al., 2009). However, their development has been remarkably little-studied at the molecular level. We were intrigued by the hypoxia- and hypercapnia-sensitivity of adrenal chromaffin cells in mammals (Comline and Silver, 1966, Cheung, 1989, Cheung, 1990, Seidler and Slotkin, 1985, Seidler and Slotkin, 1986, Thompson et al., 1997, Muñoz-Cabello et al., 2005, García-Fernández et al., 2007, Levitsky and López-Barneo, 2009) (see also López-Barneo et al., 2016), whose development, like that of glomus cells, requires the autonomic transcription factors Ascl1 (Mash1) (Huber et al., 2002, Kameda, 2005) and Phox2b (Dauger et al., 2003, Huber et al., 2005) (also see Kameda, 2014). We aimed therefore to investigate the extent to which glomus cell development might parallel adrenal chromaffin cell development. Our analyses in chicken and mouse indeed revealed significant similarities with adrenal chromaffin cell development, at both molecular and cellular levels.

### 4.1 Glomus cells and adrenal chromaffin cells develop along similar, but not identical, molecular pathways

We found that glomus cell precursors at the periphery of the developing chicken carotid body at E7.5–E9.5 were immunoreactive for the neuron-specific RNA binding proteins Elavl3/4 (HuC/D; Okano and Darnell, 1997; Pascale et al., 2008). Glomus cell precursors were previously reported to express the neuron-specific deubiquitinating enzyme PGP9.5/Uchl1, as well as neuronal βIII tubulin (Tubb3) (Kameda et al., 1993, Kameda, 1995, Kameda, 2005). Our gene expression data show that the neuronal glomus cell precursors express not only *Phox2b*, but also other known ‘sympathoadrenal’ genes (Huber, 2006, Huber, 2015, Huber et al., 2009, Kameda, 2014, Lumb and Schwarz, 2015): the transcription factor genes *Hand2*, *Gata2*, *Sox11*, *Hif1a*, *Hif2a* (*Epas1*; *Hif1a* and *Hif2a* were only expressed by a subset of neurons) and the receptor tyrosine kinase gene *Ret*. By E12.0–13.5, serotonergic glomus cells expressing Tubb3, but no longer Elavl3/4, were present in the carotid body core (with some Elavl3/4^+^ neurons still at the periphery), expressing multiple ‘sympathoadrenal’ genes. Taken together, these data suggest that neuronal glomus cell precursors expressing multiple 'sympathoadrenal' genes move from the carotid body periphery to the core, where they up-regulate serotonin and down-regulate Elavl3/4 (though not Tubb3) as they mature.

This pattern of development is reminiscent of the differentiation of adrenal chromaffin cells from neuronal precursors in the adrenal medulla, which down-regulate pan-neuronal markers, lose *Ret* expression and mature as chromaffin cells (Anderson and Axel, 1986, Anderson et al., 1991, Huber et al., 2002, Huber et al., 2005). *Ret* downregulation may be important for adrenal chromaffin cell maturation, since in *Ascl1*^*-/-*^ mice, *Ret* is maintained in the adrenal gland and the ultrastructure of adrenal medullary cells more closely resembles that of sympathetic neurons than chromaffin cells (Huber et al., 2002). Although *Ret* expression was maintained (albeit weakly) in chicken glomus cells at E12.0–E13.5, we found that glomus cell development was unaffected in *Ret*^-/-^ mouse embryos, like adrenal chromaffin cells (Allmendinger et al., 2003).

The basic helix-loop helix transcription factor Hand2 is a downstream effector of Bmps in sympathetic neuron specification (Howard et al., 1999, Howard et al., 2000, Wu and Howard, 2001), influencing both the early proliferation of sympathoadrenal progenitors and *Phox2a, Gata3* (whose expression we identified in developing mouse glomus cells) and *TH* expression in developing sympathetic neurons (Hendershot et al., 2008, Schmidt et al., 2009). A previous report suggested there were fewer *TH*-expressing adrenal chromaffin cells at P0 after *Hand2* deletion in the neural crest (Hendershot et al., 2008). Recently, the *Periostin-Cre* transgenic allele (Lindsley et al., 2007, Takeda et al., 2010) was used to delete *Hand2* almost completely from the adrenal medulla, without affecting *Hand2* expression in sympathetic ganglia (Vandusen et al., 2014). This resulted in a significant reduction at postnatal stages in the expression in the adrenal medulla of the catecholamine biosynthesis genes *TH* and *Dbh* (encoding dopamine β-hydroxylase) and the adrenaline-synthesising enzyme gene *Pnmt* (encoding phenylethanolamine-N-methyltransferase) (Vandusen et al., 2014). We found that *Hand2* is also required in the neural crest for glomus cell specification and differentiation: there was no obvious carotid body condensation in *Wnt1-Cre/Hand2*^*flox/flox*^ embryos, and neither TH nor serotonin was expressed.

The SoxC family transcription factors Sox4 and Sox11 are required for sympathetic gangliogenesis, with Sox11 most likely influencing immature sympathetic neuroblast proliferation and Sox4 promoting cell survival later (Potzner et al., 2010). *Sox11* is a direct Hand2 target (Holler et al., 2010). Deletion of *Sox4* and *Sox11* in the neural crest resulted in a hypomorphic carotid body phenotype, similar to that reported for two cervical sympathetic ganglia (the superior cervical ganglion and stellate ganglion) after deletion of these genes using a *Dbh-Cre* driver line (Potzner et al., 2010): the number of glomus cells was greatly reduced, but the remnant cells expressed serotonin, *Phox2b* and *TH*. Thus, as in the sympathetic ganglia (Potzner et al., 2010), *Sox4* and *Sox11* may be required for glomus progenitor proliferation and survival. However, unlike *Hand2*, they are not essential for glomus cell differentiation. Loss of *Sox4* and *Sox11* in the neural crest did not significantly affect the number of TH-positive adrenal chromaffin cells (although their distribution was abnormal, with some found within the cortex), in contrast to the reduction seen in the numbers of both sympathetic neurons and glomus cells.

The expression of *Hif1a* and *Hif2a* (*Epas1*) during glomus cell development suggests a role for these hypoxia-inducible transcription factors in glomus cell differentiation, not just in the functioning of the adult carotid body (Kline et al., 2002, Peng et al., 2011, Yuan et al., 2013). *Hif2a* is expressed in mouse adrenal chromaffin cells (Tian et al., 1998) and is required for the normal expression of DOPA decarboxylase and DBH (though not TH) in an adrenomedullary chromaffin cell line (Brown et al., 2009). Indeed, hypoxia promotes the catecholaminergic differentiation (expression of TH and DBH, with synthesis and release of dopamine and noradrenaline) of neural crest stem cells (Morrison et al., 2000). Thus, *Hif2a* may also be required for the catecholaminergic differentiation of developing glomus cells.

The mechanisms underlying glomus cell development are not identical to those underlying adrenal chromaffin cell development, however. In *Tfap2b*-null mice, the sympathetic ganglia are significantly reduced through neural crest progenitor death, but *TH*^+^ adrenal chromaffin cells are present in normal numbers; however, a significant reduction was noted in the expression of *Phox2b*, *Dbh* and the adrenaline-synthesising enzyme gene *Pnmt*, suggesting an effect on chromaffin cell maturation (Hong et al., 2008, Hong et al., 2011, Schmidt et al., 2011). In contrast, we found that serotonergic, *TH*^+^ glomus cells developed normally in *Tfap2b*^-/-^ mice. Furthermore, as noted above, deleting *Sox4* and *Sox11* in the neural crest had no significant impact on the number of TH-positive adrenal chromaffin cells, but there were many fewer glomus cells.

Taken together, our data suggest multiple parallels at the molecular level between developing glomus cells and developing adrenal chromaffin cells. This is consistent with our recent discovery of neural crest-derived catecholaminergic (chromaffin) cells associated with blood vessels in zebrafish gill arches, which we speculated could be homologous to the ancestral cell population from which glomus cells evolved (Hockman et al., 2017).

### 4.2 Multipotent progenitors with a glial phenotype contribute to glomus cells

Genetic fate-mapping with tamoxifen-inducible *Sox10*^*CreERT*2^ (Laranjeira et al., 2011) and *Plp1*^*CreERT2*^ (Leone et al., 2003) drivers recently revealed that, in addition to precursors segregating at the dorsal aorta (see e.g. Saito et al., 2012), a significant proportion of adrenal chromaffin cells originate from multipotent progenitors with a glial phenotype ("Schwann cell precursors") associated with the preganglionic sympathetic nerve fibres that innervate the adrenal gland, which upregulate neuronal and catecholaminergic markers (Furlan et al., 2017). This discovery has resolved many previously raised inconsistencies with the older model of sympathetic neurons migrating secondarily to the adrenal medulla from primary sympathetic ganglia, including the lack of TH expression in cells in the adrenal medulla when sympathetic neurons already express TH (Gut et al., 2005), the presence in the adrenal gland anlage of Sox10^+^ and Phox2b^+^ cells that lack TH or neuronal marker expression (Ernsberger et al., 2005), and differences in synaptic and pan-neuronal protein expression between developing adrenal chromaffin cells and sympathetic neurons (Stubbusch et al., 2013) (also see Huber, 2006, Huber, 2015; Huber et al., 2009; Lumb and Schwarz, 2015).

Our genetic lineage-tracing data using the tamoxifen-inducible *Plp1*^*CreERT2*^ driver line (Leone et al., 2003) suggest that multipotent progenitors with a glial (*Plp1*-expressing) phenotype contribute to glomus cells. Such cells could be associated with the carotid sinus nerve afferents distributed around the third pharyngeal arch artery at E12.5 (Hertzberg et al., 1994, Kameda et al., 2008), and/or they could be the precursors of the neurons in the superior cervical ganglion proposed from histological studies to migrate into the adjacent carotid body in various mammals, including mice (Kohn, 1900, Smith, 1924, Korkala and Hervonen, 1973, Kameda et al., 2002, Kameda et al., 2008, Kameda, 2005, Kameda, 2006).

### 4.3 Resolving a paradox in birds for the 'émigré' hypothesis of glomus cell precursor origins

For well over a century, histological analysis of sections of embryonic series from various mammals (including human) and, more recently, chicken, has suggested that glomus cells develop from precursors that emigrate from neighbouring ganglia and/or nerves (Kohn, 1900, Smith, 1924, Korkala and Hervonen, 1973, Kameda, 1994, Kameda, 2002, Kameda et al., 1994). Indeed, in the mouse, glomus cell development seems to depend on the presence of both the adjacent superior cervical ganglion (Fig. S1A), which provides sympathetic innervation to the carotid body, and the afferent carotid sinus nerve (Kameda, 2006, Kameda et al., 2008) (also see Kameda, 2014).

Our fate-mapping data resolve the inconsistency associated with this hypothesis in birds, where the closest ganglion to the developing carotid body is not the neural crest-derived superior cervical ganglion, as in mammals, but instead the nodose ganglion (Fig. S1B), in which satellite glia are neural crest-derived but most neurons are placode-derived (Narayanan and Narayanan, 1980, D’Amico-Martel and Noden, 1983, Kious et al., 2002). By labelling the vagal neural crest using GFP-transgenic to wild-type chicken neural fold grafts, we identified a small population of neural crest-derived neurons in the nodose ganglion and in the ‘bridge’ between the nodose ganglion and the developing carotid body, at least some of which express the autonomic marker *Phox2b*. Thus, the putative 'émigrés' from the nodose ganglion to the carotid body are indeed neural crest-derived, unlike most nodose neurons, resolving the apparent paradox for the 'émigré' hypothesis of glomus cell origins in the chicken. Furthermore, neurons in the 'bridge' express the catecholamine biosynthesis gene *TH*, supporting their identity as glomus cell precursors. These data are also consistent with previous quail-chick grafting experiments showing that neural crest-derived cells in the nodose ganglion are competent to form both catecholaminergic adrenal chromaffin cells and sympathetic neurons when pieces of nodose ganglia are back-grafted into the trunk neural crest cell migration pathway (Ayer-Le Lièvre and Le Douarin, 1982).

### 4.4 Conclusions

Overall, our data reveal significant parallels between glomus cell and adrenal chromaffin cell development at both molecular and cellular levels (though also some differences at the molecular level). They provide support for the existence of a broadly common differentiation programme for initiating catecholaminergic development in three neural crest-derived autonomic cell types: glomus cells in the neck, adrenal chromaffin cells in the mid-trunk and sympathetic neurons throughout the trunk. Different requirements for specific transcription factors or signalling pathways must separate glomus cell, adrenal chromaffin and sympathetic neuron fates: identifying such differences will be important for any attempts to use carotid body glomus cells (and/or their adult sustentacular cell stem cells; Pardal et al., 2007) for patient-specific Parkinson's disease therapy.
