# Supplementary material for: Striking parallels between carotid body glomus cell and adrenal chromaffin cell development
Source: Dev Biol. 2018 Dec 1;444(Suppl 1):S308–24. doi: 10.1016/j.ydbio.2018.05.016 (PMC6453021; doi:10.1016/j.ydbio.2018.05.016)
Supplement: Supplementary file 1 — Supplementary material [file mmc5.docx]

**Supplementary File 1: ImageJ script applied to *Plp1^CreERT2/+^;R26R^YFP/+^* mouse carotid body confocal image stacks**

//Macro written by Dominic Waithe for Dorit Hockman

//edited by Dorit Hockman

//It is assumed that CH1 is blue, CH2 is green, CH3 is red.

//run on stacks.

CH2 = 2;

CH3 = 3;

//Checks default colours are used.

run("Colors...", "foreground=black background=white selection=yellow");

//Finds input title

input = getTitle();

//Duplicates first channel

run("Duplicate...", "duplicate channels="+CH2);

//Thresholds using a static value

setThreshold(57, 255);

setOption("BlackBackground", false);

run("Convert to Mask", "method= background=Dark black”);

rename("CH2");

//Counts pixels in CH2.

out_count = 0

setBatchMode(true);

stack = getImageID();

for (slice=1; slice<=nSlices; slice++) {

if (nSlices>1) run("Set Slice...", "slice=" + slice);

getHistogram(values,counts,2);

out_count = out_count + counts[1];

}

setBatchMode(false);

print(input,"Pixels CH2 (green): \t",out_count);

//Counts pixels in CH3

selectWindow(input);

run("Duplicate...", "duplicate channels="+CH3);

//Thresholds using a static value

setThreshold(57, 255);

setOption("BlackBackground", false);

run("Convert to Mask", "method= background=Dark black”);

rename("CH3");

out_count = 0

setBatchMode(true);

stack = getImageID();

for (slice=1; slice<=nSlices; slice++) {

if (nSlices>1) run("Set Slice...", "slice=" + slice);

getHistogram(values,counts,2);

out_count = out_count + counts[1];

}

setBatchMode(false);

print(input,"Pixels in CH3 (red): \t\t",out_count);

//Creates image where colocalisation is shown as yellow

run("Merge Channels...", "c1=CH3 c2=CH2 create keep");

//Finds those pixels which are shared between both channels.

imageCalculator("AND create 32-bit stack", "CH2","CH3");

rename("out");

out_count = 0

setBatchMode(true);

stack = getImageID();

for (slice=1; slice<=nSlices; slice++) {

selectImage(stack);

if (nSlices>1) run("Set Slice...", "slice=" + slice);

getHistogram(values,counts,2);

out_count = out_count + counts[1];

}

setBatchMode(false);

//Prints output

print(input,"Pixels in Both \t\t\t",out_count);

//Cleans up some of the images.

selectWindow("CH2");

close();

selectWindow("CH3");

close();
